# The *Staphylococcus aureus* CC398 Lineage: An Evolution Driven by the Acquisition of Prophages and Other Mobile Genetic Elements

**DOI:** 10.3390/genes12111752

**Published:** 2021-10-30

**Authors:** Floriane Laumay, Hugo Benchetrit, Anna-Rita Corvaglia, Nathalie van der Mee-Marquet, Patrice François

**Affiliations:** 1 Genomic Research Laboratory, Service of Infectious Diseases, Faculty of Medicine, Geneva University Hospitals, 1211 Geneva, Switzerland; floriane.laumay@chu-lyon.fr (F.L.); anna-rita.corvaglia@hcuge.ch (A.-R.C.); 2 Institut des Agents Infectieux, Centre de Biologie du Nord, Hospices Civils de Lyon, F-69003 Lyon, France; 3 UFR de Chimie et de Biologie, Faculté des Sciences, Université Grenoble Alpes, 38000 Grenoble, France; hugo.benchetrit@etu.univ-grenoble-alpes.fr; 4 Geneva Centre for Emerging Viral Diseases, Faculty of Medicine, Geneva University Hospitals, 1211 Geneva, Switzerland; 5 UMR 1282 Infectiologie Santé Publique, Université de Tours, F-37032 Tours, France; N.VANDERMEE@chu-tours.fr

**Keywords:** *Staphylococcus aureus*, CC398, evolution, prophages, mobile genetic elements

## Abstract

Among clinically relevant lineages of *Staphylococcus aureus*, the lineage or clonal complex 398 (CC398) is of particular interest. Strains from this lineage were only described as livestock colonizers until 2007. Progressively, cases of infection were reported in humans in contact with farm animals, and now, CC398 isolates are increasingly identified as the cause of severe infections even in patients without any contact with animals. These observations suggest that CC398 isolates have spread not only in the community but also in the hospital setting. In addition, several recent studies have reported that CC398 strains are evolving towards increased virulence and antibiotic resistance. Identification of the origin and emergence of this clonal complex could probably benefit future large-scale studies that aim to detect sources of contamination and infection. Current evidence indicates that the evolution of CC398 strains towards these phenotypes has been driven by the acquisition of prophages and other mobile genetic elements. In this short review, we summarize the main knowledge of this major lineage of *S. aureus* that has become predominant in the human clinic worldwide within a single decade.

## 1. Introduction

*Staphylococcus aureus* is a ubiquitous human and veterinary pathogen recognized as a worldwide health problem. It persistently colonizes the skin of around 20% of healthy individuals but is also responsible for a wide spectrum of infections, ranging from local benign skin infections to severe disseminated diseases such as endocarditis or osteomyelitis [1,2]. In the hospital environment, the bacterium shows particular capacity to adapt its metabolism to environmental changes including antimicrobial or biocide pressures or host defenses [3]. Despite highly different clinical manifestations, the number of staphylococcal lineages involved in human and veterinary clinical infections is limited to approximately ten, accounting for the totality of infections due to *S. aureus*. Highly successful lineages rapidly emerged in the clinic showing specific resistance determinants or metabolic advantages. In this epidemiological context, strains belonging to clonal complex 398 (CC398) are a fascinating subject of interest, having emerged in the human clinic without any obvious or understandable causes.

The lineage 398 may have first been detected in 2003, in the Netherlands. The authors of the study aimed to characterize community clones of methicillin-resistant *S. aureus* (MRSA) that emerged in a few individuals involved with pig farming and that were non-typable by pulsed-field gel electrophoresis. These clones increased in proportion from 0% in 2002 to more than 20% of MRSA, in the Netherlands, in 2006. The study revealed that the density of these MRSA strains correlated with the density of pig populations, an observation consistent with human carriage being significantly related to contact with pigs or cattle. Interestingly, a large number of these MRSA belonged to a new sequence type (ST), the ST398 [4]. Around the same time, in France, a study reported that the higher rate of *S. aureus* colonization in healthy pig farmers was caused by a few STs, including ST398, found in pigs but not in non-farmers, and that most of these isolates were sensitive to methicillin (MSSA) [5]. Other studies identified ST398 strains in animals, including pigs, dogs, horses, and in humans, in Europe and in Asia [6,7,8,9,10]. Starting from 2007, cases of severe infection due to CC398 isolates were increasingly reported in different places where humans were in contact with livestock (mostly MRSA), but also in individuals without any contact with animals (mostly methicillin-sensitive) [11,12,13,14,15,16,17,18]. Analysis of whole genomes from different populations of ST398 isolates revealed quite homogeneous sequences, except for the absence/presence of specific prophages and other mobile genetic elements (MGEs), which enabled differentiation of strains responsible for asymptomatic colonization in livestock from strains involved in bloodstream infections in humans living in animal-free environments [15,16,19,20,21,22,23]. 

Annual surveillance programs allowed observation of a constant increase in the prevalence of CC398 *S. aureus* as etiologies of severe infections, especially bone and joint infections (BJIs) and bloodstream infections (BSIs) [24,25]. Recent studies show that the lineage continues to evolve in terms of virulence and resistance [26,27], and there is increased evidence that isolates have, during the last decade, acquired genetic features contributing to host adaptation and virulence through integration of prophages [28,29].

The purpose of this short review is to present the main studies of evolution of the *S. aureus* lineage 398 and its prophages and other MGE acquisitions, and our current understanding of these phenomena at the molecular level.

## 2. Clinical Importance of the CC398 Lineage

**Figure 1:**
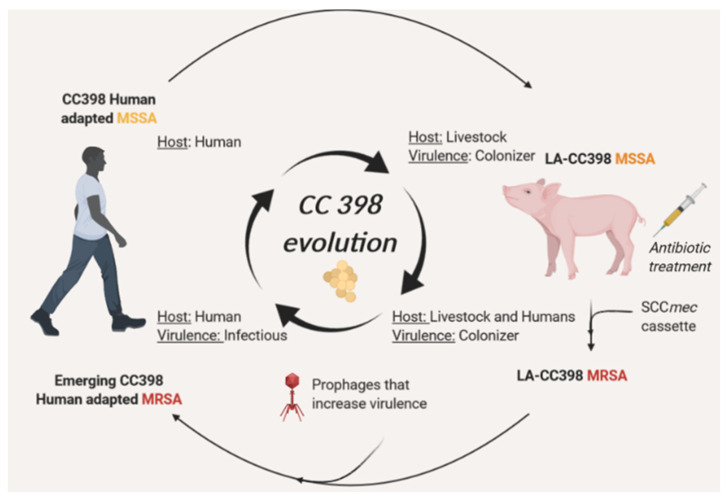
Emergence of CC398 human-adapted MRSA clade. CC398 human-adapted clade is the name of the presumed methicillin-susceptible ancestor clade. Evolution of tropism through cattle and integration of prophages/*SCCmec* element led to the emergence of the new methicillin-resistant human adapted clade.

The distribution of CC398 has evolved considerably since this CC was first detected in the early 2000s as MRSA strains colonizing asymptomatic breeders and industrially raised pigs [4,5], and later cattle, poultry, horses, rabbits and pets [30,31,32,33]. Rapidly, the human carriage and persistence of CC398 MRSA was correlated, in several countries, with exposure to colonized animals and with intensity of contacts with animals (livestock-associated (LA)-MRSA), especially in pig farmers [5,34]. Several studies have clearly demonstrated exchange of ST398 strains between animals and humans (Figure 1), such as this recent study that described the unidirectional transmission of CC398 MRSA strains from animal to human in pig-farming settings [35]. Of importance, it is well known that *S. aureus* colonization has an important impact on infection rate [1,36]. However, the literature contains only a few studies related to the prevalence of CC398 as a colonizer in the global human population. Holtfreter and colleagues published a nice study about the nasal colonization in the general population in a large region of Germany [37]. CC398 MRSA and MSSA, including LA genetic backgrounds, were detected by the authors. Note however that CC398 was only rarely found as a nasal colonizer and that characterization of these strains generally failed to detect any potent toxin (i.e., Panton–Valentine leukocidin (PVL) and enterotoxins). Intestinal colonization is perhaps more relevant for this bacterium originating from an animal environment, but studies analyzing intestinal colonization have not been done. Recently, a study published by Chiaruzzi and colleagues analyzed tampon colonization in healthy women, which might be more representative of vaginal colonization than studies based on vaginal swabbing [38], and identified CC398 *S. aureus* as a prevalent vaginal colonizer [39]. As described for CC398 strains identified as nasal colonizers, these isolates were also devoid of superantigens (enterotoxin and *tst* genes). Of note, the prevalence of *S. aureus* was higher in tampons that do not require an applicator, suggesting also a transmission route through the hands.

A number of human infections due to CC398 have also been documented following colonization by CC398 MRSA, first in European farmers and veterinarians [4,5], and then, starting from 2007 in Europe [13,17], CC398 MSSA was found in patients without any contact with animals. However, cases of infection due to MRSA and MSSA from the CC398 lineage have been described worldwide, in very distant geographic regions [11,12,16,18]. This is illustrated in the review published in 2015 by Smith and Wardyn who identified 74 publications describing CC398 infections in humans [40]. Even if the majority of CC398 infections have been reported in Europe, the review compiles studies from 19 different countries (Austria, Belgium, Canada, China, Colombia, Denmark, Dominican Republic, Finland, France, Germany, Greece, Italy, Netherlands, Norway, Spain, Sweden, Scotland, Switzerland and USA). The majority of the strains was negative for the presence of the gene encoding the PVL. Of note, the prevalence of CC398 (MSSA and MRSA) varied significantly by country. In specific regions of China, CC398 has been reported as one of the most frequent types of infectious MSSA and may be of human rather than livestock origin, since, in the scope of these studies, most MRSA from Chinese pigs belonged to ST9 [41,42]. In contrast, in the Netherlands, the prevalence of MRSA belonging to ST398 has substantially increased from 2002 to 2007 (from 0% to > 20%) and has been correlated with the density of pig herds in the country [4], a trend described in other European countries [43]. Recently, Bouiller and colleagues reviewed human infections caused by CC398 MSSA [44] and indicated that, although these strains are present in several countries across the world with a heterogeneous prevalence, the majority of CC398 infections have been reported in European countries (France, the Netherlands, Germany, Spain, Italy, Czech Republic, etc.) and Asian countries (different regions of China and Korea), but now also in countries such as United Arab Emirates and Iran. Although the PVL-encoding gene has been only rarely identified in CC398 MSSA genomes, the authors highlighted the recent spread in China of a PVL-producing subpopulation [45].

Clinical manifestations caused by CC398 strains are diverse, but according to the recent review of Bouiller et al. [44], invasive and severe infections are often caused by CC398 MSSA, whereas CC398 MRSA is mainly implicated in skin and soft tissue infections (SSTIs). Nonetheless, the authors point out the likely underestimation of SSTIs caused by CC398 MSSA because most countries do not perform MSSA surveillance, or if they do, they only focus on invasive infections. BJIs, BSIs, or bacteremia caused by CC398 are a particular matter of concern. An important study from 12 foot clinics in France reported that CC398 MSSA account for 38% of 161 cases of osteomyelitis complicating diabetic foot ulcers, showing an enrichment of the genotype in causing some diseases [25]. Another important study on almost 1000 cases of bone and joint infections associated with *S. aureus,* collected over an 8-year period, showed that the prevalence of CC398 has increased from 4% in 2010 to 26% in 2017, and that the majority of isolates was sensitive to methicillin [46]. Regarding BSIs, for example, in France, the first cases of BSIs caused by CC398 *S. aureus* were detected in 2007, including in patients who had no contact with farm animals; ten years later, almost 15% of the total BSIs reported in France was caused by CC398 MSSA [13,24,47]. In China also, CC398 belongs to predominantly MSSA genotypes isolated from bacteremia [48,49]. 

## 3. Prophage Content of the CC398 Sub-Populations, Phage-Encoded Factors

Originally described as colonizing asymptomatic pigs and pig farmers, the CC398 lineage has undergone two major epidemiological changes. First, the host range of CC398 has been extended to other livestock and pet species, but also to humans living in animal-free environments. Second, CC398 virulence has progressively increased, since isolates from this lineage are now a primary cause of BSIs due to *S. aureus* in some countries [13,24,44,48,49], and are responsible for severe invasive infections such as endocarditis and osteomyelitis [25,44,46]. These changes yielded three distinguishable clades within the CC398 lineage: (i) the classical livestock-associated (LA) clade responsible for asymptomatic colonization and rare infection in animals, farmers and veterinarians; (ii) the ancestral human clade, which is also LA; and (iii) the emerging human-adapted CC398 clade that is responsible for an increasing number of invasive infections worldwide in individuals without any contact with animals. Despite distinct phenotypic features, these strains remain “clonal”, with little variation in the core genome, but differ on the basis of their MGE content. Prophages in particular appear to play a key role in the evolutionary changes of the CC398 lineage [15,16,21,22]. This is consistent with studies conducted with *Streptococcus pyogenes* and *Streptococcus agalactiae* that have argued that prophages may play a role in the emergence of lineages with increased capacity to infect humans and animals [50,51].

Indeed, comparative genomics studies did not identify any gene of the core genome as essential for host specificity (e.g.*,* same variants of the *isdB*, *ssl-10*, *fll* and *flr* genes between pig and human isolates) whereas prophage content appeared to be specific to each CC398 sub-population. The prophages φ2 and φ6 are frequently found in both pig and human LA-CC398 isolates; conversely, the presence of β-converting φ3-prophage variants carrying an immune evasion cluster (IEC) characterizes the emerging human-adapted clade. Infecting isolates also harbor MR11-like prophages and other prophages from the Sa3int group [21,22,23]. Of note, integration/excision of β-converting Sa3int prophages was reported to play a role in host adaptation of several *S. aureus* lineages (CCs 9, 5, 97, 8 and 121 notably), as recently reviewed by Rohmer and Wolz [52]. Indeed, human to animal transmission is strongly correlated with the loss of Sa3int prophages [15,16], but it seems that LA-CC398 MRSA are able to readapt to the human host through regain of an IEC-harboring Sa3int prophage [53,54,55]. 

Regarding CC398 phage-encoded factors, numerous genes are associated with greater virulence or immune evasion in animal models of staphylococcal infections. The IEC of prophage φ3 is composed of genes encoding for immune-modulating proteins: the chemotaxis inhibitory protein of *S. aureus* (CHIPS) that blocks neutrophil recruitment, a staphylokinase (*sak*) which is an inhibitor of defensins, and the staphylococcal complement inhibitor (SCIN) that prevents opsonophagocytosis and killing of *S. aureus* by human neutrophils [21,56,57]. Additionally, the IEC acquisition is associated with increased household transmission of LA-CC398 MRSA and spillover into the community and healthcare settings [55]. The φ3 variants also carry genes encoding putative factors that have been shown to be involved in bacterial virulence, biofilm formation, fitness and adaptation to challenging environments, and resistance to foreign DNA uptake (e.g.*,* leukocidin-like protein, small RNA SprD, tyrosine recombinase XerC, ATP-dependent Clp protease, LexA/antirepressor protein KilAC-mediated mechanism and HNH endonuclease). Similarly, MR11-like prophages have been shown to carry genes encoding an ADP-ribosyltransferase toxin, a putative phage defense mechanism, an autolysin Atl, a putative SsrA-SmpB toxin–antitoxin system, a glycolytic operon, and a putative superantigen similar to enterotoxin B. Additional elements have been found in other prophages (e.g.*,* virulence-associated protein VirE, lysostaphin-like glycyl-glycine endopeptidase, ALE-1, bacterial Ig-like domain, putative restriction modification systems, type III secretion system YopX) [22,24,58,59,60,61,62,63,64,65,66,67,68]. Expression of some of these genes has been documented under conditions known to induce the transcription of phage-encoded virulence factors via the activation of the SOS system (i.e.*,* stressful situations such as exposure to sub-lethal concentrations of antibiotics). Interestingly, an association between φ3 and MR11-like prophages may favor the expression of the IEC genes, an observation already reported in the association between a β-converting phage and a helper phage [22,69,70,71].

## 4. Roles of CC398-Borne Prophages in Bacterial Virulence

CC398-prophages have been documented to play a role in escaping the host immune response, invasion of epithelial cells, adhesion to host cells and extracellular matrix components, and production of toxins contributing to an increased ability of the bacteria to colonize and infect the host. 

Regarding host immune escape, expression of immune-modulating proteins encoded by β-converting prophages contributes to long-term colonization in humans [57,69,72]. Of note, prophage disruption of staphylococcal virulence genes would limit the human immune response and favor bacterial colonization, and perhaps subsequent infection [73]. In addition, CC398 isolates harboring β-converting prophages have been shown to be less efficiently phagocytosed by human and equine neutrophils; however, the presence or the absence of the prophage did not seem to matter for phagocytosis efficiency with porcine neutrophils [74]. Note that to exclude an isolate effect, the authors included in their study three isogenic φSa*int*3 negative/positive strain pairs obtained following mobilization of a β-converting prophage (with *sak*, *chp* and *scn* genes) from a chicken LA-MRSA CC398 isolate into φSa*int*3-negative LA-MRSA isolates derived from cattle and turkey. The authors suggested that IEC component-mediated immune escape is not specific to human species and that the absence of protective effects against phagocytosis by porcine neutrophils linked to the presence of φSa*int*3 might explain why these prophages are only rarely found in pig-borne CC398 isolates. Otherwise, although not an obligatory intracellular pathogen, *S. aureus* virulence, particularly in chronic infections, has been correlated with its ability to survive within the cytoplasm of non-phagocytic host cells, in which it is protected from immune host defenses and antimicrobials [75,76,77]. The capacity of representative CC398 isolates to invade epithelial cells has been associated with the presence of φ3 variants [22]. In addition, Gerlach and colleagues showed that a large proportion of LA-CC398 MRSA harbors prophages (φ*tarP*-Sa1int and φ*tarP*-Sa9int) encoding an alternative glycosyltransferase called TarP that is able to substitute for the activity of its bacterial homolog TarS [78]. Both enzymes glycosylate wall teichoic acids (WTAs), though differently, and modification of WTAs by TarP attenuates WTA immunogenicity and results in a decrease in anti-WTA antibody production in mice and human sera.

Bacterial adhesion is another property important for *S. aureus* pathogenesis. The adhesins are bacterial cell wall surface proteins that allow interactions with host extracellular matrix components, and thus, colonization of host cells and tissues; these molecules have also been shown to be essential for invasion of endothelial and epithelial cells in an experimental model of infections [75,79,80,81,82,83,84]. Uhlemann and colleagues demonstrated that human ST398 MSSA isolates bind to keratinocytes and purified keratin (from human and porcine origins) more than LA-MRSA ST398 isolates. Importantly, the two sub-groups harbored distinct MGEs (various integrative conjugative elements (ICEs), *S. aureus* pathogenicity islands (SaPIs), φ2 and φ6 prophages), but also varied in their repertoire and composition of adhesion genes [16]. Recently, our group compared bacterial adhesion and invasion, but also *in vivo* virulence of isolates belonging to different CC398 sub-populations [28]. In the study, a representative panel of CC398 isolates was collected, including two “ancestral” animal isolates devoid of any prophages, and “model strains” that did or did not contain prophages were produced, similarly to what Jung and colleagues did. We showed that strains with prophages (StauST398-2Pro and StauST398-3Pro) have acquired different properties related to virulence such as an increased capacity to adhere to human fibrinogen and fibronectin-coated surfaces, which correlated with an increased capacity to invade non-phagocytic cells. In addition, all strains containing prophages were more invasive than the prophage-free parent strain in a model of infectious endocarditis in rats. Interestingly, we found that the expression of the genes mainly responsible for adhesion to human fibronectin and fibrinogen (*fnpbA* and *clfA*, respectively) was higher in the lysogens. Of note, Bonesso et al. also reported differences in expression levels of the α-toxin, phenol-soluble modulin peptides and protein A between ST398 and non-ST398 isolates responsible for pneumonia. This observation was corroborated by the increased capacity of ST398 to lyse human erythrocytes and neutrophils, and to cause severe and multifocal necrotizing pneumonia in a mouse pneumonia model; all ST398 isolates were MSSA and contained the *chp* and *scn* genes, but the authors did not further investigate their MGEs content [85]. Another recent study has shown that ST398 virulence in a *Caenorhabditis elegans* infection model was not solely explained by genomic type, but rather by the presence of φSa3 and its structural variants [29]. 

A summary of the main CC398-borne prophages and their respective roles in bacterial virulence is presented in Table 1.

## 5. Continuous Evolution of CC398 towards Highly Virulent and Resistant Phenotypes through MGEs Acquisition

A number of recent studies report an increase in virulence and resistance in CC398 isolates. Sieber and colleagues performed comparative genomics to elucidate the drivers of evolution and the evolutionary dynamics of the LA-MRSA CC398 isolates. They highlighted CC398 as an evolving clade resistant to nearly all β-lactams, several non-β-lactam antimicrobials, and heavy metals. An increased bacterial resistance, which in combination with pig movements between farms and pig trading, has resulted in an uncontrolled spread of CC398 over the years [27,86], an observation extended to spread via other animals [87,88]. In MSSA clones, this spread is not limited to a specific geographical area but is common even between very distant sites, and is not limited to agricultural regions, as it is also described in large cities such as New York and the surrounding area [89,90,91]. The latter study described strains exhibiting resistance to clindamycin and erythromycin. Bouiller and colleagues showed that CC398 MSSA spreads not only in the community but also in hospital settings—similar to the famous USA300 clone observed in the US—and described current bloodstream infections involving CC398 “as marker of fatal outcome” [26]. 

These phenotypic changes have been partially attributed to the acquisition of additional MGEs, including SCC*mec* cassettes, transposons, and plasmids harboring antimicrobial resistance genes [21]. CC398 isolates continue to acquire new MGEs or accumulate known MGEs with structural variations [92,93]. Notably, a recent review described various small plasmids, which can integrate/be integrated into larger plasmids or within the bacterial chromosome. Individually, these plasmids encode generally for 1–4 resistance genes and are key players in dissemination of certain antimicrobial resistance genes among LA-MRSA ST398 and even across animal species, bacterial species, and geographic regions [94]. These different plasmids have been shown to carry the *erm(C)* or *erm(T)* genes conferring resistance to macrolide, lincosamide, and streptogramin B antibiotics; the lincosamide resistance gene *lnu(A)*; the pleuromutilin-lincosamide-streptogramin A resistance genes *vga(A)* or *vga(C)*; and the genes *spd*, *apmA* and *dfrK*, respectively procuring the resistance to spectinomycin, apramycin and trimethoprim. Recently, Ruiz-Ripa and colleagues described, for the first time in Spain, LA-MRSA CC398 carrying the linezolid resistance gene *cfr* [95]. Of particular concern is the increasingly frequent detection of CC398 isolates carrying multiple determinants of virulence and resistance in food products that can be the vehicles for transmission of clinically important *S. aureus* strains [96,97]. Recently, Li and colleagues described, for the first time, CC398 strains isolated from Danish foods carrying the *tst* gene encoding the toxic shock syndrome toxin 1 (TSST-1), which probably originated from the acquisition of a SaPI-like element [96]. Mama et al. analyzed more than 100 pig-derived food samples collected in Northern Spain and found that 23% of *S. aureus* isolates were LA-MRSA CC398, which were almost all multidrug resistant (more than 82%) [97]. 

## 6. Conclusions

The *S. aureus* CC398 lineage has become a major public health problem in only a few years. Originally detected in pig breeding, the current farming practices, combined with massive utilization of antibiotics, have resulted in the progressive dissemination of CC398 in different host species, including humans, and across geographic boundaries; these CC398 isolates exhibit very different virulence and resistance profiles. Many studies attributed host tropism, virulence, and resistance properties of CC398 isolates to acquisition of MGEs, in particular prophages such as φ3 and MR11-like, which have been shown to increase *in vitro* virulence potential and *in vivo* virulence in an experimental model of infections. Of importance, several recent articles warn that this lineage continues to acquire new MGEs, and will evolve in terms of virulence and resistance. Overall, these findings should encourage efforts to limit spread and to identify key steps of this evolutionary process that can inform infection-control strategies.

## Figures and Tables

**Table 1:** Roles of the main CC398-borne prophages in bacterial virulence.

Prophages	Characteristics	Putative roles	References
φ3 and Sa3int variants	β-converting, IEC, putative factors involved in bacterial virulence, biofilm formation, fitness, stress adaptation and genome plasticity	Immune escape	[21,22,56,57,74]
		Host colonization, invasion	[22,24,58,59,60,61,62,63,64,65,66,67,68]
		Long-term host colonization	[57,69,72,73]
		Virulence in *Caenorhabditis elegans*	[29]
MR11-like variants	Putative factors involved in bacterial virulence, biofilm formation, fitness, stress adaptation and genome plasticity	Host colonization, invasion	[22,24,58,59,60,61,62,63,64,65,66,67,68]
		Immune escape, when associated to φ3	[22,69,70,71]
φ*tarP*-Sa1int and φ*tarP*-Sa9int	Alternative glycosyltransferase TarP	WTAs modification, immune escape	[78]
StauST398-2Pro and StauST398-3Pro		Adhesion to fibronectin/fibrinogen—modulation of adhesion genes expression, cell invasion, virulence in rats (infectious endocarditis)	[28]
